# Thermal bridging in buildings: A critical review of mechanisms, mitigation strategies, energy impacts, and research needs

**DOI:** 10.1177/01436244261453333

**Published:** 2026-05-14

**Authors:** Malcolm Ahsan, Maged A. Youssef, Chris Van Dongen, David Thompson, Greer Stanier

**Affiliations:** 1 Department of Civil and Environmental Engineering, 6221 Western University, London, ON, Canada; 2 Entuitive, Toronto, ON, Canada

**Keywords:** thermal bridging, building envelope, energy performance, thermal performance, structural thermal breaks

## Abstract

The increasingly stringent energy and emission targets have made thermal bridging a critical consideration for building construction. Thermal bridging is excessive, localized heat flow through discontinuities in the insulation layer, such as at window and door assemblies, building envelope junctions, or balconies, that significantly increases space-heating and cooling demand, depresses interior surface temperatures, and promotes moisture-related durability problems. This paper presents a state-of-the-art critical review of thermal bridging in buildings, with emphasis on its mechanisms, quantification, and mitigation. The classifications, causes, and locations of thermal bridges within the building envelope are synthesized, and methods for evaluating them, including the Equivalent Wall Method (EWM), the Equivalent U-Value Method (EUVM), and 2D/3D numerical modelling, are compared. Element-specific mitigation strategies for openings, exposed structural components, walls, junctions, and balconies are reviewed, highlighting their influence on effective envelope R-values, building energy use, and condensation risk. The literature shows that thermal bridges account for a substantial fraction of heat loss in highly insulated buildings and become proportionally more important as envelope requirements tighten. Key knowledge gaps are identified, including the scarcity of long-term experimental validation and the need for holistic, constructible solutions that integrate structural, architectural, and thermal performance.

**Practical Application:**

Thermal bridging can materially affect heating and cooling loads, interior surface temperatures, and moisture risk in high-performance envelopes. This review provides building professionals with a comprehensive foundation to identify high-impact details (openings, slab edges, balconies, and junctions), select appropriate quantification methods (Ψ/χ values, EUVM, and 2D/3D modelling) for design and compliance, and prioritize constructible mitigation strategies, such as, but not limited to, continuous insulation, thermally broken connections, and improved detailing and quality control. The review supports a more holistic, durability-focused decision-making process through efficient HVAC system sizing and smart envelope design.

## Introduction

Climate change, population growth, and rising energy costs have pushed building sustainability to the forefront of public policy. Buildings and the construction industry account for about 30–40% of global energy and materials use and approximately 40–50% of greenhouse gas emissions.^1^ The majority (80–90%) of a building’s total energy use occurs during its operational phase,^2^ mainly for space heating and cooling, domestic hot water, and lighting. In cold climates such as Canada, energy use is dominated by space heating; for example, in 2017, about 63% of energy used by the residential and commercial sectors went to heating and cooling, accounting for 17.5% of Canada’s total energy use.^3^ Additionally, researchers predict that global energy demand will increase by 50% by 2050,^4^ underscoring the urgency to improve building energy efficiency and sustainable construction practices.

Building codes and standards, such as ASHRAE or the Canadian National Energy Code, impose minimum energy performance requirements.^5,6^ However, many jurisdictions adopt stricter local regulations, such as the Toronto Green Standard^7^ and Vancouver’s Zero Emissions Building Plan.^8^ Owners or designers looking to exceed expectations typically explore sustainability programs such as LEED^9^ and Passive House,^10^ which aim to achieve further reductions in building energy demand, particularly for heating and cooling, among other sustainability-driven design targets.

A significant, yet underappreciated factor influencing building energy consumption is thermal bridging. A thermal bridge is typically defined as a localized area of the building envelope where heat flows more readily due to an interruption or reduction of the insulation layer.^11^ Common examples include window frames, slab edges, and structural elements that penetrate the insulation, such as balcony slabs. If left unmitigated, thermal bridges increase heat loss in winter and heat gain in summer, forcing HVAC systems to work harder to maintain comfort, increasing their energy demand, and potentially creating condensation, mould, and durability issues. While thermal bridging mitigation is not explicitly mandated in most North American codes, increasingly stringent performance targets make it indispensable, especially when considering effects outside of energy demand.

This paper presents a state-of-the-art review of thermal bridging in buildings. It synthesizes current knowledge on definitions and fundamentals, typologies and locations, methods for quantifying thermal bridges, their impacts on energy performance and moisture risk, and mitigation strategies. It also identifies key research gaps and future directions relevant to energy-efficient building design and retrofit.

## Scope and review methodology

This paper is a critical narrative review of thermal bridging in buildings, focusing on both fundamental understanding and practical design implications. The literature was identified through searches of major scientific databases. The review emphasizes peer-reviewed journal papers, standards and technical reports, and selected references from industry design guides. Studies published between approximately 2010 and 2025 form the core of the synthesis, with earlier foundational work included where necessary to explain methods and definitions. The purpose of the review is to provide readers with a baseline understanding of thermal bridging (what it is, how it forms, where it occurs, why it is an issue, and how it is analyzed and mitigated), and to highlight potential future research directions. Therefore, no meta-analysis or statistical aggregation of the available datasets has been provided, as it falls outside the scope and purpose of the review. Due to large variability in performance targets and the highly nuanced, dependent nature of the performance metrics, it is extremely difficult to normalize results across studies and to create a generic framework for evaluating a structure’s thermal and energy performance.

The paper is structured to move from fundamentals to applications. Section 3 introduces the fundamental concepts and definitions used throughout the review, including the sensitivity of thermal bridges to climate. Section 4 reviews the mechanisms and typology of thermal bridges by building element, with emphasis on openings, exposed structural components, walls, junctions, and balconies. Section 5 summarizes methods used for quantifying thermal performance, how thermal bridges affect these calculations, and how to incorporate these into an overall performance metric, before discussing the tools used to quantify performance metrics and linear and point thermal transmittance values. Section 6 synthesizes current knowledge on the impacts of thermal bridges on energy performance, condensation risk, and thermal comfort, including results from dynamic simulations and payback analyses. Section 7 reviews mitigation strategies by element, linking analytical findings to practical detailing solutions. Section 8 identifies research needs and future directions. Lastly, Section 9 summarizes the main conclusions of the review.

## Fundamentals and definitions

### Thermal performance metrics

Several thermal performance metrics are used throughout this review. The U-value (thermal transmittance) describes the rate of heat transfer through a material or assembly per unit area and per degree of temperature difference (W/m^2^·K); lower U-values indicate better insulation and reduced heat loss. The R-value (thermal resistance) is the inverse of the U-value (m^2^·K/W) and expresses resistance to heat flow; higher values indicate better insulating performance. It should be noted that the R-values mentioned throughout the review are in units of (^0^F·ft^2^·hr/BTU), which is 5.678 times the metric equivalent, as this is the industry standard in North America. Thermal conductivity (λ) is an intrinsic property of a homogeneous material (W/m·K) that quantifies heat flow per unit thickness (*t*) and temperature difference and, unlike U- or R-values, does not depend on geometry. A single material has an R-value equal to *t* divided by λ and, therefore, a U-value equal to λ divided by *t*.

**Figure 1.**
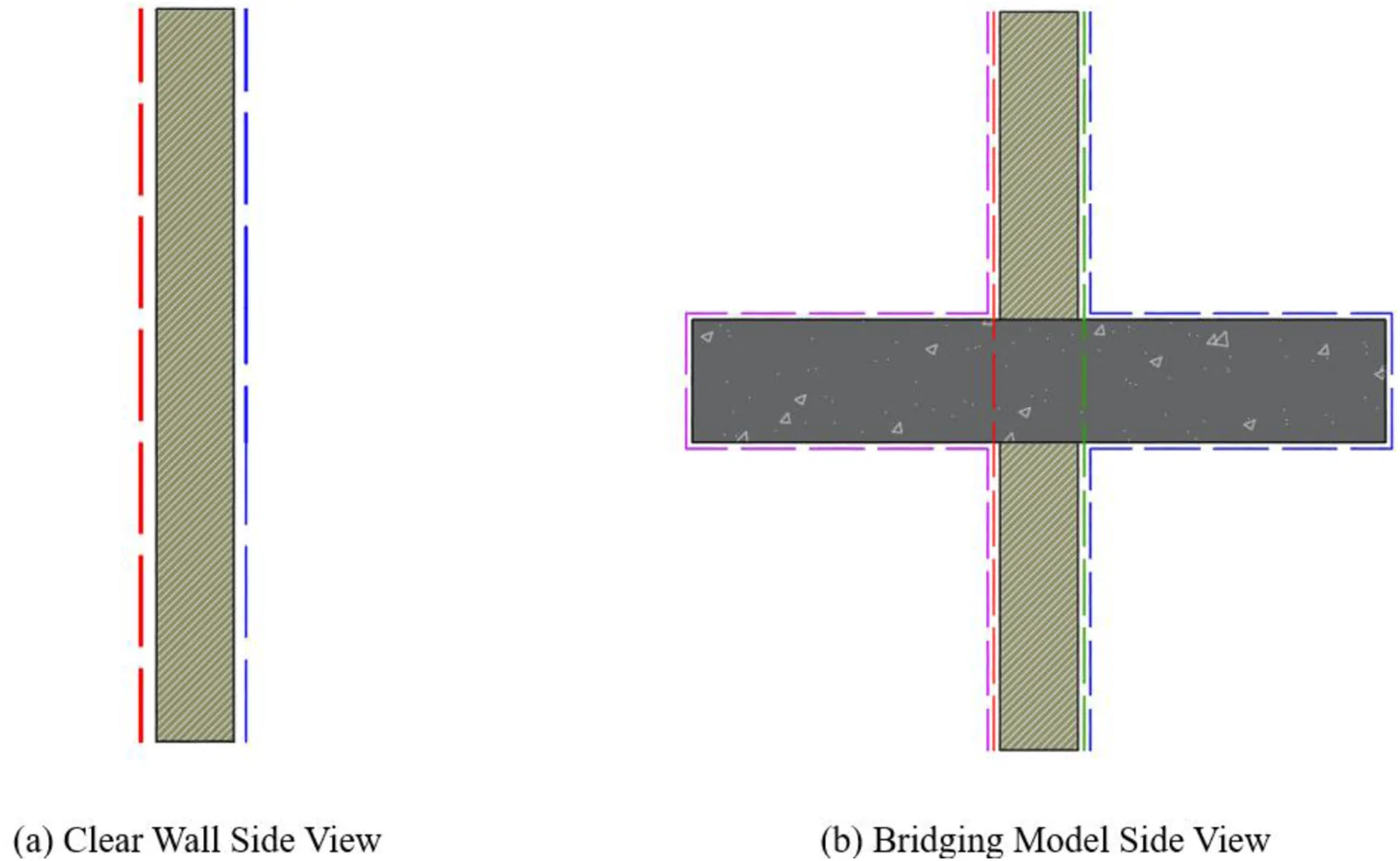
Wall schematics comparing the geometry of a clear wall with a thermally bridged wall.

A clear wall is an idealized wall with continuous insulation and no thermal bridges; it serves as a baseline against which bridging effects are measured. Figure 1 schematically compares a clear wall with a thermally bridged wall. Thermal bridges are often described as either linear or punctual. A linear thermal bridge extends along a line in the envelope, such as a balcony slab, slab edge, or window perimeter, and is characterized by a linear thermal transmittance Ψ-value (psi-value) in W/m·K, which represents the additional heat flow per unit length caused by the bridge relative to the clear wall.^11^ A punctual (point) thermal bridge is localized at a point, such as a metal fastener, anchor, or wall tie penetrating insulation. It is characterized by a point thermal transmittance χ-value (chi-value) in W/K.^11^ These Ψ- and χ-values provide a convenient way to quantify and compare the additional heat flow associated with various details. They can also be used to predict the impacts of thermal bridges on the envelope.

### Climate sensitivity

Thermal performance metrics such as U-, R-, Ψ-, and χ-values are defined per degree of temperature difference and, in principle, are independent of climate. However, the actual heat flow through a thermal bridge is directly proportional to the indoor–outdoor temperature difference, ΔT. Consequently, the same detail can have vastly different impacts in different climates.^12^ Table 1 demonstrates this, showing the heat flow, Ψ-value, and effective R-value of a typical concrete balcony slab analyzed at several outdoor temperatures, with a constant indoor temperature of 21.5°C. Although the Ψ-value and effective R-value remain nearly constant at about 0.78 W/m·K and R-8.9, respectively, the heat loss per metre (Q_AVG_) increases more than fivefold as the exterior temperature falls from +15°C to −15°C (ΔT increasing from 6.5°C to 36.5°C). In cold climates such as Canada or Northern Europe, a given thermal bridge contributes a much larger share of the heating load than in milder climates. It can create a condensation risk due to the gradient resulting from a large ΔT between the interior and exterior surfaces. In very hot climates, the same bridge may instead drive unwanted heat gains and increased cooling loads. Climate thus does not change the intrinsic thermal properties of a bridge. However, it significantly affects outcomes, especially in highly insulated buildings, where the bridges’ relative importance is amplified.

**Table 1.** Thermal performance of a balcony thermal bridge under different climate conditions.

Int. Temp. (^0^C)	Ext. Temp. (^0^C)	Q_AVG_ (W/m)	Ψ-Value (W/m·K)	Effective R-value
+21.5 (indoor)	+30 (hot outdoor)	11.95	0.7807	8.889
	+15 (mild outdoor)	9.134	0.7807	8.889
	0 (freezing)	30.19	0.7796	8.896
	−15 (very cold)	51.25	0.7796	8.896

Throughout the review, thermal bridging effects will be discussed using Ψ/χ Values or their impact on overall thermal performance (i.e., effective U/R-value). It should, however, be noted that looking solely through the lens of thermal performance is not sufficient in all cases for 2 main reasons. Firstly, different climates lead to different primary issues caused by thermal bridging. This factor will be discussed further in Section 4. The second, and more pressing, reason is that the codes and standards engineers use do not prescribe a U-value. Typically, codes will prescribe limitations on the Total Energy Use Intensity (TEUI), Thermal Energy Demand Intensity (TEDI), and/or Greenhouse Gas Emissions (GHG). Practitioners can either design the building and check it against the limits, or use these values to back-calculate an acceptable envelope U-value and design the envelope accordingly. However, this is not a simple procedure, as the TEDI, TEUI, and GHG consider more than just thermal performance, depending on the envelope and superstructure geometry and physical properties, as well as the climate, electrical systems, and mechanical systems. For this reason, instead of discussing definitive performance targets, the review will focus on the impacts on the decision-making process for designing and analyzing structures.

## Mechanisms and typology of thermal bridges in buildings

Thermal bridging arises from the interaction of geometry, materials, and construction practice, and it is convenient to classify bridges by both cause and physical extent.

### Causes and configurations

Three principal classes of thermal bridges are commonly cited in the literature^11,13^ and are grouped by their formation mechanisms. The classes are Geometric thermal bridge, Structural (or Construction) thermal bridge, and Systematic (or repeating) thermal bridge.

**Figure 2.**
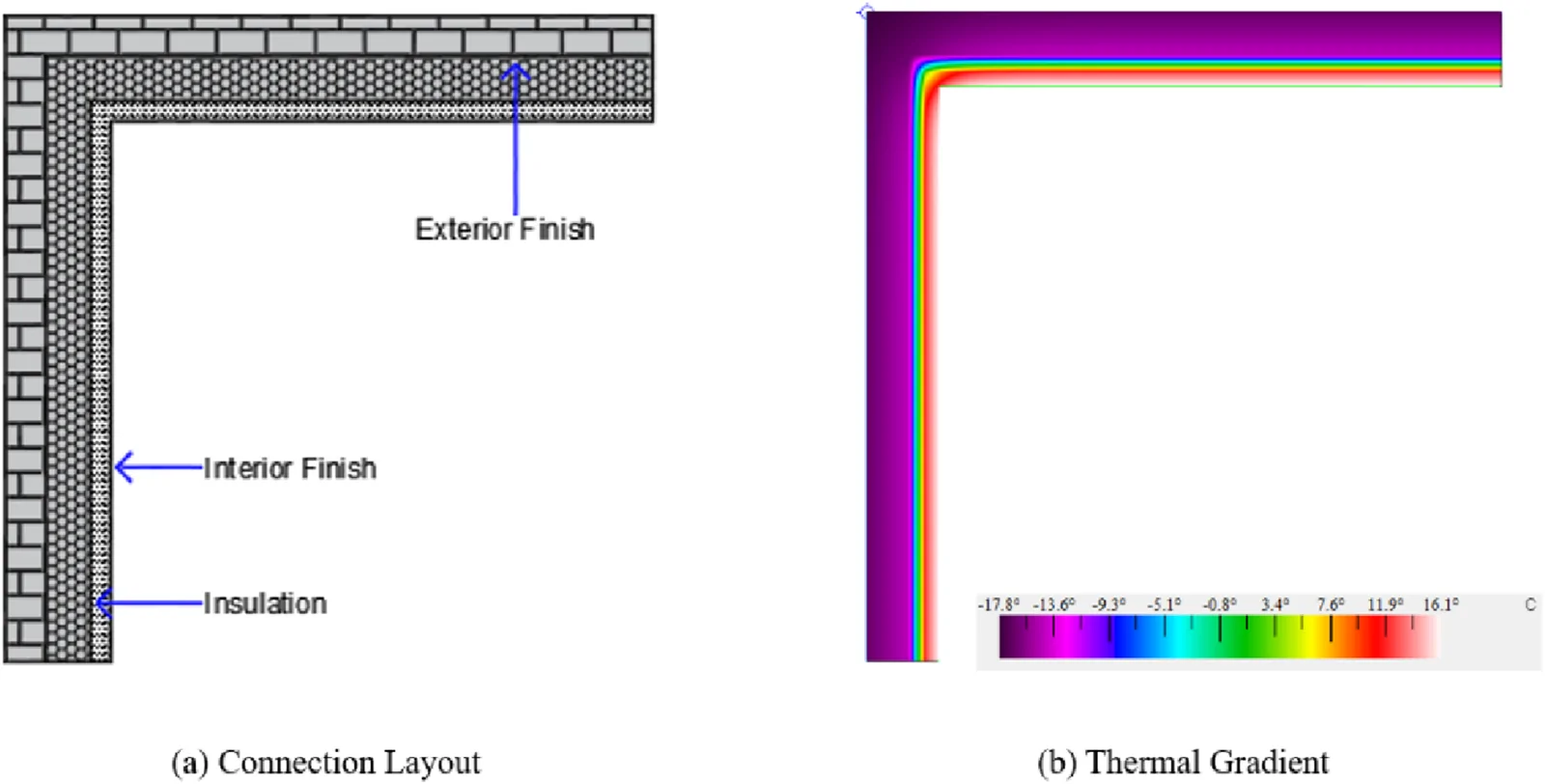
Geometrical thermal bridge at an exterior corner illustrating intensified heat flow.

Geometrical thermal bridges arise from the envelope’s shape rather than from material changes. At corners and junctions, such as wall-to-wall or wall-to-roof intersections, the external surface area can exceed the internal area, so that even with continuous insulation, heat flow is locally intensified. Figure 2 illustrates a wall-to-wall corner where the external surface area around the corner exceeds the internal area, leading to higher local heat loss and lower interior surface temperatures.

**Figure 3.**
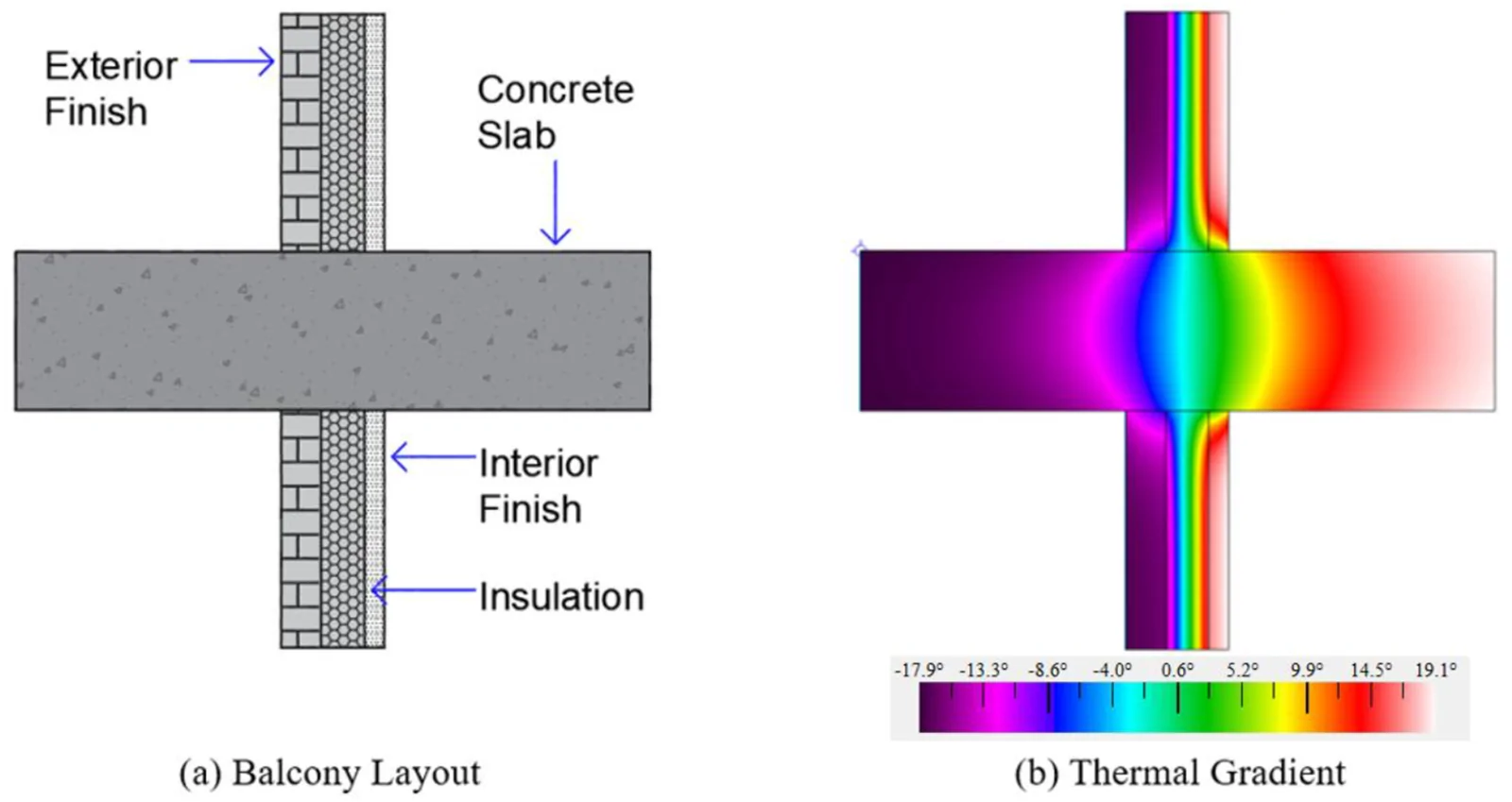
Structural thermal bridge caused by a conductive element penetrating the insulation layer.

Structural, or construction, thermal bridges occur when structural or envelope components penetrate, bypass, or replace insulation. Typical examples include concrete floor slabs extending as balconies, steel beams, openings (windows and doors), or parapets that cut through the thermal barrier (Figure 3). These elements are essential for structural and functional reasons, but create direct conductive paths.^12^

**Figure 4.**
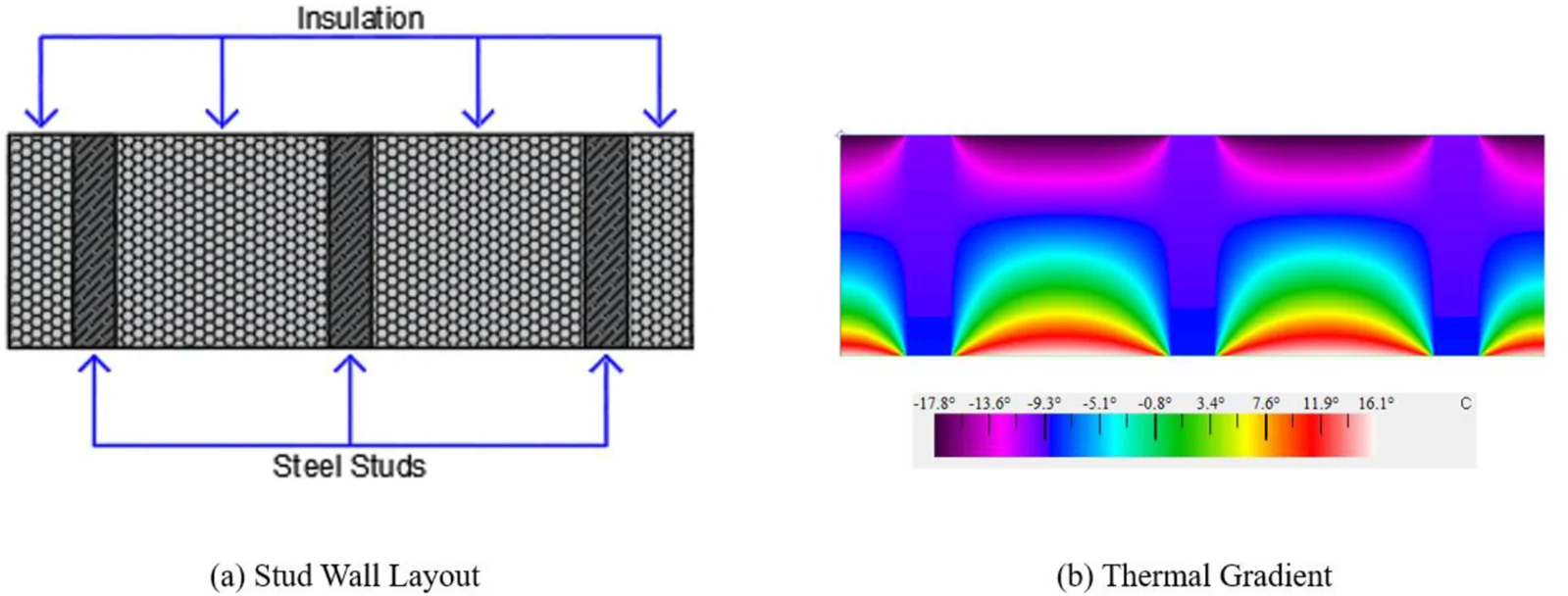
Systematic (repeating) thermal bridge due to regularly spaced conductive fasteners or studs.

Systematic, or repeating, thermal bridges arise from repetitive components such as metal studs, brick ties, and cladding attachment clips that pass through insulation at regular spacing (Figure 4). Individually, each bridge may be small, but the repeating pattern of bridges can significantly reduce the assembly’s effective R-value,^11^ especially if the spacing is low or studs/anchors are highly conductive.

From a geometrical perspective, thermal bridges are also distinguished as linear or punctual.^14^ Linear bridges, such as slab edges, balcony–wall junctions, or window perimeters, extend along a line and are characterized by their Ψ-value. Point bridges, such as anchors, bolts, and discrete bracket supports, are localized and characterized by their χ-value. In practice, details often involve a combination of geometric, structural, and systematic effects, with both linear and point bridges forming.

At a basic level, all thermal bridges can be mitigated by increasing insulation around the bridging elements, thereby inhibiting excessive heat flow. However, it is important to understand how different types of thermal bridges form so their impacts can be assessed and the most effective mitigation strategies applied. Regardless of the location of the thermal bridge, mitigation strategies for a particular class of bridge will hold commonalities since the underlying cause of the bridge is the same. For example, structural thermal bridges occur through penetrations in the insulation layer. A common practice to mitigate these bridges is to add thermal isolation material between the joints. This occurs in many areas, such as balcony slabs, penetrating beams, and columns connecting to the foundations. Also, the extent (linear vs punctual) of the thermal bridge affects how one analyzes the assembly and what simplifications can be made (e.g., modelling a single typical section of a repeating punctual bridge or modelling 1m of a large linear bridge to get a per-meter measurement). Lastly, commonalities between how bridges are incorporated into envelope-level calculations can be connected back to the types of thermal bridges. Systematic thermal bridges, like wall studs, tend to work as a modification to the wall’s U-Value, whereas structural bridges, like balconies, tend to be incorporated into the calculation through their Ψ/χ-value.

### Openings

Openings are among the most significant and frequent thermal bridge locations in building envelopes.^15^ Replacing a high-R wall section with a lower-R window or door directly increases heat flow through the opening, creating a large, punctual, structural thermal bridge with linear thermal bridges around its perimeter, through the frames and supporting elements (headers, sills, jambs). The magnitude of heat flow at openings depends on glazing configuration, frame material and geometry, and installation quality.^16,17^ The greater the disparity between the thermal performance of the wall and that of the window or door assembly, the more pronounced the bridging effect, caused by increased heat flow directly through the opening and the severity of linear thermal bridges around it.

Experimental and analytical work has highlighted the importance of both glazing and frames. Upgrading from single to multi-pane low-e glazing with inert gas fills can dramatically lower glass U-values,^18^ reducing the magnitude of thermal bridging through the frames. However, poorly performing frames can erode much of this benefit. High-conductivity aluminum frames, if not thermally broken, can dominate losses, whereas less conductive frames, such as wood, uPVC, or fibreglass, can retain the thermal benefits of other design choices.^18,19^ Smusz et al.^20^ quantified the effect of insulated versus open-profile PVC frames. Table 2 summarizes key findings from their study. They showed that insulated frames offered modest but consistent reductions in the thermal coupling coefficient (L_2D_) and linear transmittance (Ψ) at typical connections, with the largest relative benefit at the sill. L_2D_ is the total heat flow, divided by the bridge length and ΔT, describing how well heat flows through an assembly.

**Table 2.** Thermal performance of window-wall connections with standard versus insulated frames.

Wall connection	Frame Type	L_2D_ (W/m·K)^20^	% difference	Ψ-Value (W/m·K)^20^	% difference
Sill	Standard	0.49519	3.6%	0.0343	9.6%
	Insulated	0.47661		0.0310	
Jamb	Standard	0.49937	3.1%	0.0385	1.2%
	Insulated	0.48367		0.0381	
Lintel	Standard	0.52588	3.3%	0.0650	3.6%
	Insulated	0.50829		0.0627	

Construction practice can also strongly influence the realized performance of designed openings. Gaps around frames, misalignment, and distortions can introduce unaccounted-for thermal bypasses and air leakage, leading to as-built performance that deviates from design predictions.^17,20^ Poor air sealing or installation gaps around frames degrade performance and promote moisture ingress, further amplifying the negative effects of thermal bridges. Additionally, window placement can significantly affect the assembly’s thermal performance, as thermal bridging is minimized when the window is aligned with the existing wall insulation. Any material in the opening provides more insulation than having no material above the envelope insulation layer. This concept is consistent with thermal bridge mitigation strategies, which typically perform best when they provide a continuous insulation layer along the building envelope.

Compared to many other bridging locations, openings are very large, vastly increasing the magnitude of heat flow through them. They are also highly repeatable throughout the structure, due to their necessity for function (i.e., doors in and out of the structure) and the built environment (i.e., windows for lighting and connection to the outdoors), resulting in a very high overall impact on the envelope. The thermal performance of an opening is heavily dependent on many factors, including size, material properties (opening material & supports), geometry, insulation levels, alignment, and proper installation.

### Exposed elements

Exposed elements such as sunshades, parapets, exposed columns, and slab edges frequently introduce thermal bridges. Exterior sunshades, or brise-soleil, while beneficial for reducing summer solar gains, typically consist of metallic or concrete elements that penetrate the façade insulation and act as thermal fins, conducting heat outwards in winter. In cold climates, unbroken sunshades therefore cause linear thermal bridges to form, and their net annual effect depends on the balance between summer cooling savings and winter heating demand.^11^ The impact of these elements should not be overlooked, as they can directly affect window performance. Windows are among the largest contributors to thermal bridging losses, making them among the first to be considered for mitigation. An improperly designed sunshade can undermine the effectiveness of any mitigation strategies or efficient design choices.

**Figure 5.**
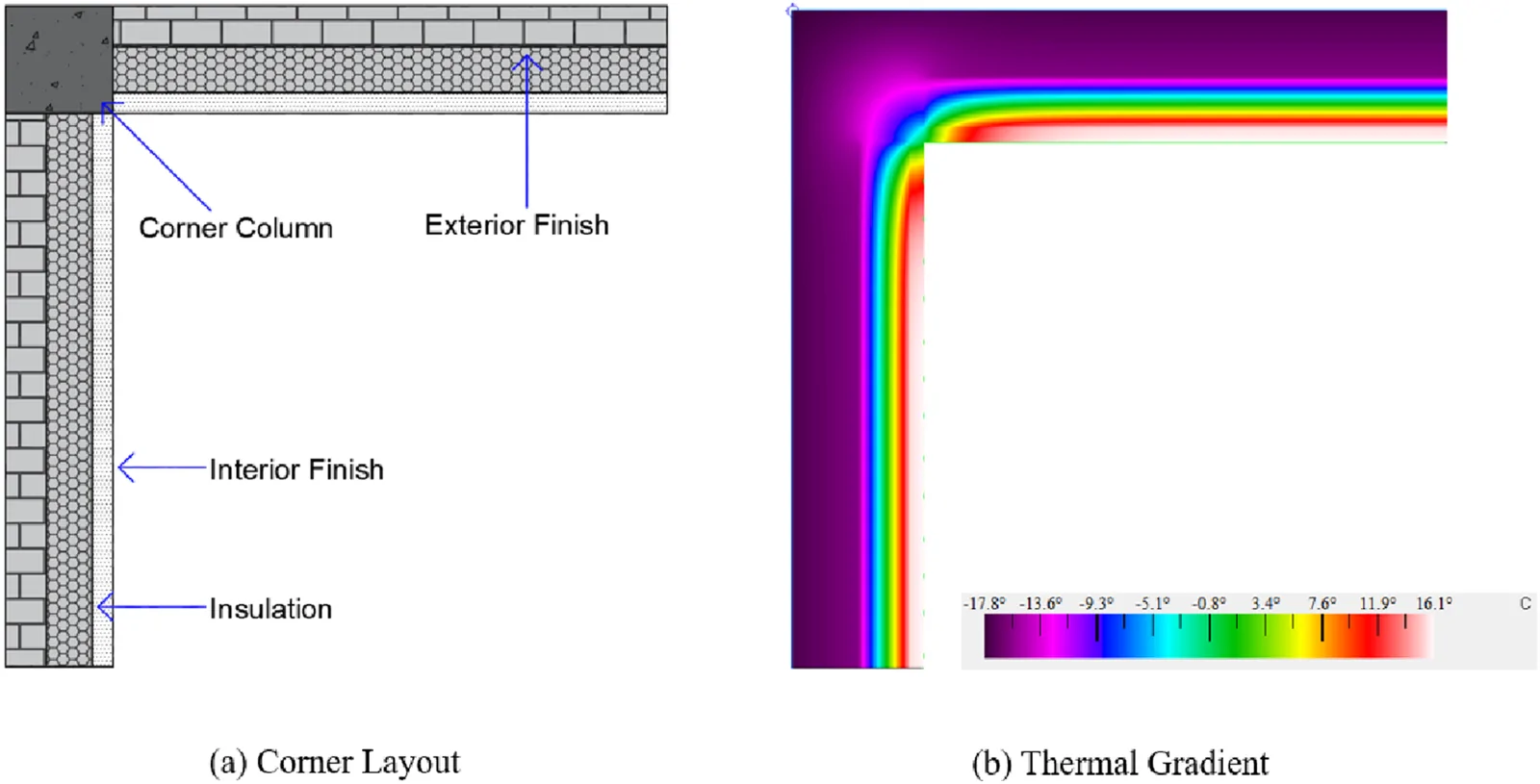
Thermal bridging at an exposed concrete corner column.

Exposed structural columns provide multiple faces for heat exchange and can significantly increase local heat loss. Jiang et al.^21^ investigated the thermal bridge of a concrete column separating a cold refrigeration room and a warm space. Due to the replacement of insulation material with an exposed structural material, these elements create a punctual, structural thermal bridge. They found that steel reinforcement, comprising only about 2.4% of the column cross-section, carried 42–56% of the total heat flow, illustrating how a small fraction of highly conductive material can dominate overall heat transfer. Exposed corner columns (Figure 5) in building envelopes can increase local heat loss by a few percent in poorly insulated walls and up to 8% in well-insulated walls.^22^ The corner column case is particularly detrimental to performance since it is subject to a geometric thermal bridge at the wall-wall connection.

Similarly, exposed slab edges occur where slabs are not wrapped in insulation or where walls sit atop slab edges rather than enclosing them. Finch et al.^23^ reported that an uninsulated 0.6 m slab overhang reduced the effective R-value of the adjacent wall section by up to 62%. Another study^12^ showed that increasing balcony length beyond about 0.5–0.8 m yielded diminishing increases of heat flow, indicating that most heat escapes near the envelope and demonstrating that a large enough slab edge, such as the 0.6 m slab edge used by Finch et al.,^23^ is analogous to a balcony, in terms of heat flow.

Although each exposed element may appear minor, its cumulative effect across multiple floors and façades can be substantial. Like openings, these elements are often highly repeatable and tend to be typical envelope details (e.g., corner columns are not placed at only one corner, sunshades are not placed over only one window, etc.), meaning their thermal impacts must be carefully considered when used in designs. This observation is especially true in high-R envelopes, where such details dominate remaining heat-loss pathways, thereby reducing the impact of envelope improvements.

### Walls

Walls make up most of the envelope area; therefore, systematic thermal bridges within them can have a large aggregate effect. This observation is particularly critical, since the wall’s R-value serves as the base value for the envelope (i.e., the clear wall). In framed walls, wood studs are moderately conductive, and steel studs are highly conductive, so the effective R-value of a stud wall is significantly lower than the nominal insulation rating. For insulated concrete sandwich panels, Sorensen et al.^24^ showed that non-composite panels with limited low-conductivity ties have negligible bridging. In contrast, partially or fully composite panels with numerous steel connectors exhibit significant point bridging and reduced effective R-values. Solid concrete regions around openings, often introduced for reinforcement or attachment, further degrade local thermal performance. These examples illustrate the problem of systematic thermal bridges; they have low individual impact but substantial cumulative impact.^25^

Construction quality is equally critical. Gaps in exterior sheathing, misfitted insulation, or moisture in insulation all act to reduce actual performance and can create narrow but continuous thermal bridges via conduction, convection, or radiation. Because wall type and layering influence the performance of other elements, walls must be considered as part of an integrated system. For example, the effectiveness of balcony thermal breaks depends strongly on their position relative to the insulation layer; misalignment or poor coordination between the break and the insulation layer can substantially reduce their benefit.

### Wall-to-wall or wall-to-roof connections

**Figure 6.**
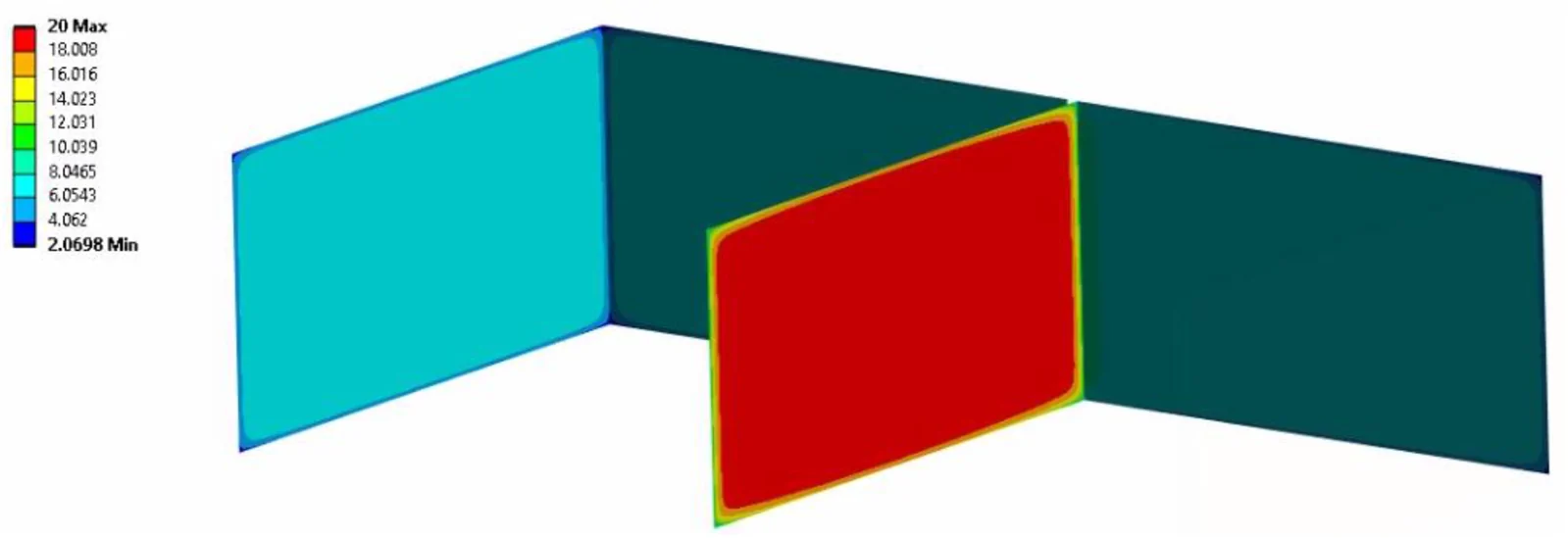
Wall–wall junction without insulation, showing a steep thermal gradient and a low interior surface temperature.

**Figure 7.**
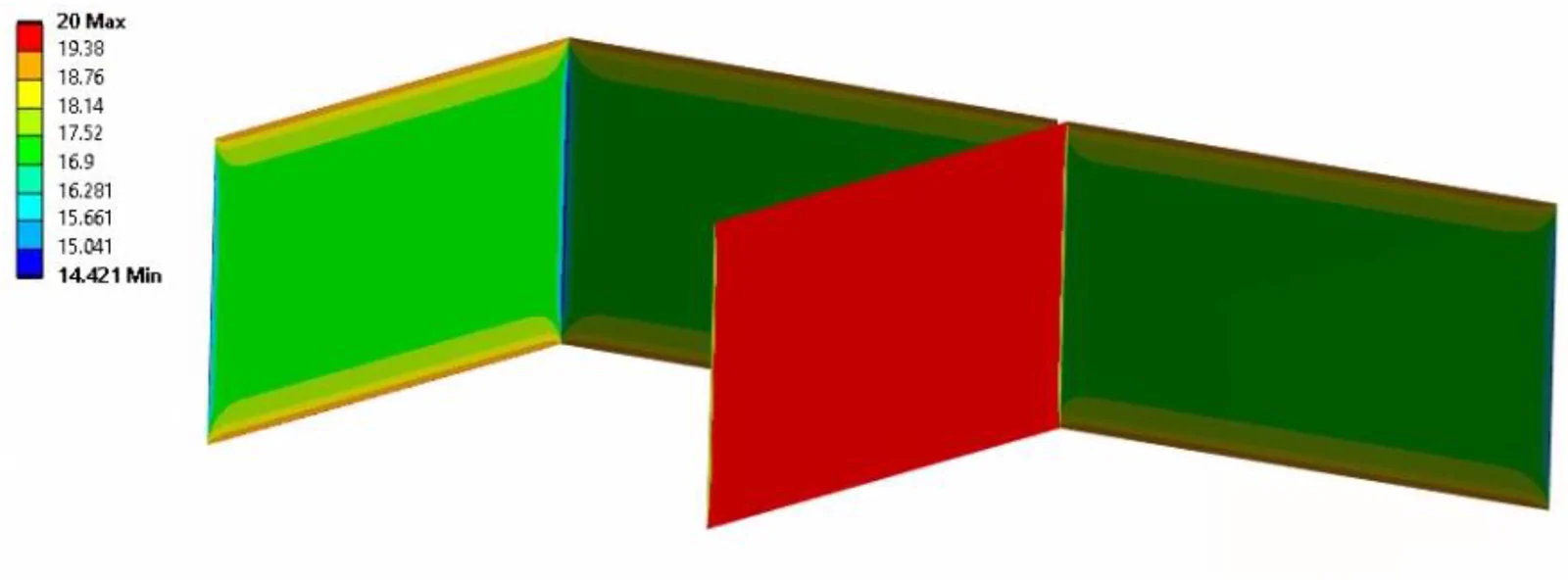
Thermal image of an insulated wall–wall junction demonstrating improved surface temperature uniformity.

Wall-to-wall and wall-to-roof connections are classic examples of geometric thermal bridges.^11^ Even with nominally continuous insulation, corners and junctions create local intensification of heat flow; any discontinuity or poor overlap in insulation at these locations exacerbates the effect. Figure 6 shows a wall–wall–ceiling corner with one interior wall and no insulation, where the thermal gradient is steep and interior surface temperatures near the junction are notably low. Figure 7 demonstrates how adding insulation at the same junction produces much more uniform temperatures and eliminates the risk of condensation. Also, the coldest temperature in Figure 7, approximately 14.4°C in the corner, is higher than any temperature seen in the exterior walls of Figure 6. If ceilings or floors remain uninsulated while walls are upgraded, substantial thermal bridging can persist at these connections. Effective design, therefore, requires coordinated insulation of all connecting planes.

### Balconies

After openings, balconies are often the most severe thermal bridges in modern multi-unit buildings.^15^ A typical concrete cantilever balcony forms a continuous linear bridge across the insulation layer, while steel balconies, supported by penetrating brackets or beams, create a series of point bridges. Finch et al.^23^ showed that balcony-induced bridges can reduce a wall section’s effective R-value by 12–60%, with the relative impact increasing as wall insulation improves. Extending the balcony length beyond about 0.5 m has a limited additional thermal effect because most heat loss occurs near the envelope interface,^12^ indicating that balcony size is seldom a critical factor for thermal performance; any non-thermally broken balcony will have a large impact on the building’s performance. Steel balconies require particular attention because steel’s high conductivity means that relatively small penetration areas can carry substantial heat. Table 3 summarizes a study by Häberli and Collins^26^ that compared various balcony configurations, including thermal-bridge mitigation strategies, for a clear wall with R22 insulation. They showed that the continuous unbroken slabs reduced the R-value by 59%. However, discrete supports (balconies) connected to the floor slab through small, low-conductivity connections and structural thermal breaks (STBs) substantially improve effective R-values and reduce vertical heat loss compared to unbroken slabs.

**Table 3.** Thermal bridge mitigation strategies for a balcony.

Balcony type	Ψ-Value (W/m·K)^26^	Effective R-value (^0^F·ft^2^·hr/BTU)^26^	Vertical heat loss^26^
Continuous concrete slab (no break)	1.059	R9.1	51%
Continuous slab + wrapped insulation	0.496	R12.6	34%
Continuous slab + STB	0.252	R15.1	21%
Discrete connections (minimal slab)	0.089	R17.5	8%

### Discussion

When designing and assessing the building envelope, it is insufficient to focus on a single section or to ignore the cumulative effects of small elements. Openings, such as windows and doors, represent the most severe thermal bridging element, followed by balconies. Compared to other thermal bridge locations, these elements create large, continuous (for balconies) or highly repeatable (for openings) discontinuities in envelope insulation, resulting in a very high overall impact. Though there are many differences and nuances among the types and locations of thermal bridging, they interact with one another when evaluating the overall envelope performance. One reason is that the magnitude of the thermal bridging effect is directly related to the relative difference in thermal performance between the materials in proximity. The same exposed slab edge reduced an assembly R-value from R3.4 to R3.0 (12% reduction) and from R21.4 to R8.2 (62% reduction),^23^ illustrating that the relative impact of a thermal bridge becomes much more severe as clear-wall performance improves. Also, the building envelope is very interconnected, since load pathing and proper sealing must be considered. This leads many elements to be connected through supporting elements, such as screws or support beams (e.g., cladding attachment, window supports), or through direct conduction (e.g., exposed elements such as sunshades). All such interconnections reduce overall performance due to weak links (i.e., low-conductivity materials). This is especially prevalent when there are many small but conductive elements, such as with some cladding systems, since the accumulation of punctual bridges should not be overlooked.

One commonality across all forms of thermal bridging is that construction practices and proper installation are vital to the envelope’s overall performance. Improper installation can lead to air leakage or moisture ingress, which in turn degrades the envelope, worsens performance, and promotes the negative effects of thermal bridging, such as staining and mould growth. Misalignment of insulation layers or the use of mitigation strategies alters the heat-flow pathing from the design, drastically reducing expected performance by allowing discontinuities to form along the envelope.

Lastly, it should be noted that focusing solely on the impact on thermal performance does not account for all cases, as factors such as climate can vary. Though the temperature difference normalizes the thermal performance metrics, so the climate does not affect the evaluation of sectional properties, the climate does affect the magnitude of the heat flow, the locations/causes of thermal bridges, and the primary issues caused by thermal bridges. In colder regions, the primary issue with thermal bridging is the impact the magnitude of heat flow has on the structure’s energy demand and on occupant comfort through uneven room temperatures, such as colder floors near balconies/exposed edges and colder air temperatures near exterior walls. In warm climates, heat flow is lower, but the dew point is higher, leading to moisture-related issues as the primary cause. If the climate is warm and humid, these issues are exacerbated by increased ambient moisture and the difference between the internally controlled humidity and external ambient humidity. In such a climate, installation issues that cause air/moisture leakage are especially detrimental to the envelope’s overall performance. Especially in sunny climates, concerns about solar heat gain can affect window material selection and the decision to use a sunshade, further complicating design considerations.

Thermal performance analysis of structures requires a holistic approach that considers a multitude of internal and external factors. Standards of the location and goals of the designers (i.e., minimum compliance vs Passive House) will determine the necessary design targets. At the same time, the climate will dictate which thermal bridging effects will be most detrimental, and the specifics of the structural and envelope design will determine the location and magnitude of the bridges. By pulling all these factors together, practitioners can effectively analyze a structure and determine which areas are causing the most harm and which strategies offer the greatest benefit, while balancing against factors such as material availability and costs.

## Methods for quantifying thermal bridges

Given that thermal bridges involve multidimensional heat flow, simple one-dimensional layer-by-layer calculations are often insufficient. In practice, thermal analysis is typically carried out at the detail level and then integrated into an overall assessment of envelope performance. This section provides a concise overview of the principal analysis methods, explains how different types of thermal bridges are incorporated into envelope-level performance calculations, and summarizes the main classes of simulation tools used in practice. Owing to the complexity and project-specific nature of these analyses, the discussion is intended as an overview for practitioners rather than a prescriptive design guideline.

### Thermal analysis methods

One of the earliest methods, the Equivalent Wall Method (EWM), models a complex 2D or 3D heat flow situation using an equivalent 1D multi-layer wall. Proposed by Kossecka,^27^ the method employs structure factors and thermal capacity to capture transient heat storage^28^ and to model heat flow at the inner and outer surfaces. However, in the presence of strongly 2D or 3D heat flow, such as at junctions or balconies, the EWM becomes less accurate because a single equivalent 1D resistance cannot fully represent the complex geometry.^28^ Aguilar et al.^29^ proposed modifications for a wall–floor slab junction by partitioning heat flow between slab surfaces and introducing an effective slab heat capacity, improving model fidelity for that case. EWM can be useful for approximate analyses, but for highly three-dimensional bridges, other methods are generally preferred.

A later and widely used steady-state approach, the Equivalent U-Value Method (EUVM), modifies the clear wall U-value by accounting for individual thermal bridge impacts to provide an effective envelope U-value. It expresses the equivalent U-value $U_{eq}$ , as^11^:

(1) $$U_{eq}=U_{0}+\frac{\overset{n}{\underset{i=1}{\sum }}\Psi_{i}*l_{i}+\overset{m}{\underset{j=1}{\sum }}\chi_{j}}{A_{0}}$$

where $U_{0}$ is the clear-wall U-value, $\Psi_{i}$ is the linear thermal transmittance of bridge i with length $l_{i}$ , $\chi_{j}$ is the point thermal transmittance of the bridge j , and $A_{0}$ is the area of the clear wall.

Any additional heat flow through linear and point bridges is converted into an equivalent increase in U-value over the area. EUVM serves as the basis for many code procedures and energy models for accounting for thermal bridges. It captures total steady-state heat flow accurately when reliable Ψ- and χ-values are available, although it does not directly describe local surface temperatures or time-dependent behaviour. These transient or dynamic thermal effects will be discussed in Section 6.

### Inclusion of thermal bridge effects in calculations

Openings are analyzed using combinations of area-weighted U-values and linear transmittance values. Standards such as ISO 15099,^30^ implemented in tools like THERM,^31^ provide procedures to calculate a window’s overall U-factor by considering glass, frame, and perimeter losses, represented by the subscripts g, f, and p, respectively:

(2) $$U_{\text{window}}=\frac{U_{\text{g}}\cdot A_{\text{g}}+U_{\text{f}}\cdot A_{\text{f}}+\Psi_{\text{p}}\cdot L_{\text{p}}}{A_{T}}$$

where $A_{T}$ is the total window area and $\Psi_{\text{p}}$ represents linear losses through the frame elements.^30^

The resulting window U-factor condenses the bridging effects into a single value, which can be used directly in wall U-value calculations or models. When local surface temperatures and condensation risk must be assessed, 2D simulations of the frame–wall region are usually employed.

Exposed elements are treated according to their size and frequency. For small, regularly repeated items such as cladding clips or ties, their combined effect can be “smeared” across the wall using EUVM by adding an equivalent ΔU derived from χ and the spacing,^15^ similar to the inclusion of the window U-value to the overall envelope U-value. Larger or unique linear elements, such as a continuous sunshade or shelf angle, are typically modelled in 2D to obtain their Ψ, which is then added to the envelope’s U-value via equation (1). Major elements with complex 3D geometry may warrant explicit 3D modelling, especially if their local effects (e.g., on surface temperatures or fluxes) are critical.

Uniform walls without significant bridging can be analyzed using conventional 1D methods by summing the layer resistances. When framing, connectors, or embedded elements introduce systematic bridging, methods such as the parallel-path or isothermal-plane approaches, or code-provided correction factors (e.g., for steel-framed walls) are used to derive an effective wall U-value.^13^ Typically, these effects modify the clear wall U-value before they are included in an EUVM calculation. In more advanced practice, 2D simulation tools such as the THERM model a representative wall section including studs, sheathing, insulation, and finishes; the computed U-value inherently accounts for systematic bridging and can be used directly in energy models.

Junctions (wall-wall or wall-roof connections) are analyzed using either 2D approximations or 3D simulations. A vertical or horizontal section through a corner can be modelled in 2D to determine the linear transmittance Ψ of the junction. For more accurate results, particularly when geometry changes along the third dimension, 3D analyses of finite sections of the junction can be performed, and Ψ or χ is derived by subtracting the clear-wall heat flows from the total heat flow. Although EWM adaptations exist for the transient analysis of corners,^29^ in routine design, an EUVM with Ψ-values obtained from simulation or catalogues is more common.

Balcony thermal bridge effects are usually quantified via EUVM. Continuous concrete balconies are modelled in 2D sections to derive a linear Ψ. Discrete support systems, such as steel brackets or posts, are better represented in 3D to obtain point χ values that can be converted to equivalent linear terms if regularly spaced. Manufacturers of STB modules often provide Ψ-values for specific assemblies, and designers frequently perform paired analyses—with and without thermal breaks—to quantify improvements in effective U and energy performance.^26,32^ Determination of the Ψ- or χ-values often requires 3D thermal simulations.

Looking through the EUVM frame, some thermal bridges (openings, systematic wall thermal bridges, exposed elements, etc.) are included by modifying the clear wall U-value. In contrast, others (geometric thermal bridges at junctions, balconies, elements such as shelf angles, etc.) are considered through the Ψ- and χ-values. By pulling all thermal bridges into a single equivalent U-value calculation, the individual impacts of a bridge or a group of bridges can be assessed, allowing designers to prioritize the most impactful bridges when resources are limited.

### Overall effective thermal performance of the envelope

BC Hydro provides a spreadsheet and guide^33^ for incorporating all thermal bridge effects into the effective envelope performance. The guide provides step-by-step instructions for implementing their tools, background information on the tools’ creation and governing equations, and a typical performance range for elements (based on their Ψ/χ-value). Their spreadsheet uses the EUVM, in accordance with ASHRAE standards^34,35^ and ISO 14683,^36^ to determine the overall performance of a building and/or its sections. Practitioners must first split up the building into sections. This can be floor-by-floor or by grouping common floors. Then, all elements of the envelope, and their geometric and material properties, must be defined. Through these values, an effective, clear wall value can be calculated, including all aspects such as wall layering, framing, windows, and attachment clips. The thermal bridging effects (Ψ/χ-values) must be assessed using external software, either 2D or 3D, depending on the element/assembly in question. All these factors are combined into a single large calculation that computes the envelopes Ueq and the heat flows for each element and bridge. By assessing individual heat flows, designers can determine which elements make the greatest overall contribution to thermal losses/gains, so their efforts can be directed towards addressing the most detrimental bridges.

It is important to consider that designing using such tools is an iterative process. Once an overall perspective of the base building’s performance is established, different thermal bridging mitigation strategies or wall assemblies can be assessed, which involves creating additional sectional models to determine reduced Ψ/χ Values. The reduced Ψ/χ Values can be entered into the spreadsheet to see the improved performance and compare the benefits with factors such as cost. However, as was discussed in Section 4, improving certain sections will make the detriments of other sections worse. This necessitates creating multiple iterations or configurations to determine the optimal design.

Additionally, this process is time-consuming, and the calculations and models used to determine Ψ/χ Values are unique to the building in question. Since envelope configurations are unique and rarely repeated across structures, this process must be restarted for each building. Complex buildings will require many assembly/sectional models to determine the thermal bridging effects to be included in the overall spreadsheet, further increasing time and computational demand. Tools for performing such simulations will be discussed in the following section.

### Tools for analysis

A range of software tools is available. Two-dimensional finite element programs such as THERM^31^ or HEAT2^37^ are efficient for planar sections of moderate complexity and are widely used in practice. Three-dimensional finite element tools, including ANSYS Mechanical^38^ and HEAT3,^37^ are employed for details with inherently 3D heat paths, such as thermally broken balconies, complex junctions, or highly irregular geometries. Computational fluid dynamics (CFD) tools like ANSYS Fluent^39^ and Star-CCM+^40^ are reserved for cases where coupled conduction–convection–radiation or airflow in cavities must be simulated, for example, in ventilated façades or when natural convection strongly influences heat transfer. Ahsan et al.^12^ compared these tools (Tables 4 and 5). First, they validated against experimental data, then conducted a case study comparing a typical cast-in-place (CIP) balcony with a thermally broken (TB) balcony. They showed that 2D tools perform adequately for conventional balconies, but for thermally broken details with strongly 3D heat flow, 3D FEA and CFD provide much closer agreement with experimental reference data, whereas 2D tools under-predict heat flow. The small, consistent difference between the 3D FEA and CFD results across both the validation and case study indicates that the cause was likely a slight difference in the software’s internal functions, such as surface treatments, mesh generation, or governing equations.

**Table 4.** Comparison of thermal analysis tools to a thermally broken balcony experiment.

Software	Q_AVG_ (W)^12^	Deviation from reference (%)^12^
Reference exp.^41^	20.060	-
THERM (2D)	17.988	10.3% (lower)
ANSYS mechanical (3D FEA)	19.991	0.3% (lower)
StarCCM+ (3D CFD)	19.579	2.4% (lower)

**Table 5.** Software results for a conventional (CIP) versus thermally-broken (TB) balcony.

Balcony type	Software	Q_AVG_ (W)^12^	Deviation vs. 3D baseline (%)^12^
CIP	ANSYS mechanical (3D)	55.465	-
	THERM (2D)	53.937	2.8% (under)
	StarCCM+ (3D)	54.455	1.8% (under)
TB	ANSYS mechanical (3D)	37.981	-
	THERM (2D)	29.965	21.1% (under)
	StarCCM+ (3D)	37.162	2.2% (under)

Practitioners must choose analysis tools according to both the geometric complexity of the detail and the purpose of the assessment. Two-dimensional FEA tools are efficient for highly planar assemblies and well-suited for rapid, iterative studies. However, as the thermal path becomes more strongly three-dimensional, their accuracy declines because additional geometric simplifications are required. Three-dimensional FEA tools generally provide higher fidelity for such cases, but at the cost of greater modelling effort and computational time. On large projects, this increase in time can add up quickly. A combined approach is likely the most time-efficient way to analyze a range of thermal bridging elements across the structure. For highly planar elements or those with low 3D variability, such as stud walls or window-wall sections, 2D FEA tools will quickly and accurately assess thermal bridging effects. For more complex configurations where 2D FEA would be insufficient, such as a thermally broken balcony, or areas where 3D geometry is crucial, such as a wall-wall-roof connection, employ 3D FEA tools, which sacrifice time for accuracy. Wherever possible, standard configurations should be used so that the same Ψ/χ Values can be used.

One final notation in this section is the required detail in such simulations. The case study by Ahsan et al.^12^ uses a single material (R20) throughout the wall. If the materials’ R-value equals the wall’s Req and the wall has the same overall thickness, then the total heat flow through the wall will be accurate. However, this does not accurately predict hot spots or other, higher-order thermal effects occurring within the wall. Depending on the design or research requirements, more detail may be needed. For instance, in a more humid environment where moisture-related issues are a concern, a single-material wall would not be sufficient, as it cannot accurately predict when and where the dew point occurs within the wall. Understanding the potential issues that may arise and the simulation’s goals are crucial to choosing the appropriate analysis tool.

## Impacts on energy performance, condensation risk, and comfort

Throughout this review, the envelopes’ performance has been discussed in terms of effective R-value and energy demand, but only thermal analysis has been considered. However, the codes and standards designers follow do not limit thermal performance; they limit energy demand and intensity. The following section provides a brief overview of energy performance analysis, how moisture-related concerns are considered in the analysis, and how return on investment can affect decision-making in the industry.

### Energy demand and envelope performance

Thermal bridges can account for a large fraction of total heat loss in modern, highly insulated envelopes. Several studies have reported that thermal bridges may account for 30–40% of heat loss in well-insulated homes.^42,43^ Mitigating major bridges yields significant improvements. For example, adding STBs to balconies has been shown to increase local wall R-values by up to 66% and reduce building heating energy demand by roughly 10–15%.^26,32^ Finch et al.^23^ observed that addressing slab edges increased the effective envelope R-value from approximately R-9 to R-12 (about a 32% improvement) and, when included in whole-building analysis, reduced annual energy use by about 14%.^23,32^ These findings demonstrate that neglecting thermal bridges can lead to substantial underestimation of heating (and, in warm climates, cooling) loads. It should be noted that the overall impact of thermal performance improvements on energy demand depends not only on the magnitude of the improvement but also on the proportion of the envelope that is comprised of the bridging element. For example, mitigating balcony thermal bridging has a larger impact on energy demand if the balconies cover 100% of the perimeter, compared to if they only covered 50% of the perimeter, even though the Req for a typical section (usually based on a 1 m width to save on computation) is consistent.

### Dynamic thermal effects

Most methods described above are steady-state, meaning they assume the element/assembly/structure is kept in a consistent environment until all elements reach thermal equilibrium; no further temperature changes will occur. However, real buildings are subjected to time-varying boundary conditions, energy-powered climate control systems, and internal gains. Thermal bridges interact with daily and seasonal temperature swings, solar radiation, and HVAC control strategies. Dynamic thermal effects refer to the combined effect of the interactions among all these factors (i.e., material, environment, geometry, ventilation controls, etc.). Ge and Baba^42^ compared steady-state EWM and EUVM calculations with year-long dynamic simulations for a low-rise residential building in Phoenix (warm climate) and Quebec City (cold climate), at low and high insulation levels (Table 6). They found that steady-state methods generally under-predicted both heating and cooling loads, that the relative contribution of thermal bridges increased with higher insulation levels, and that the EUVM, in particular, could significantly misjudge cooling loads in some scenarios (with an under-prediction of about 26% in one cold-climate case). These results indicate that dynamic effects and peak conditions amplify the impact of thermal bridges beyond what simple steady-state models suggest, particularly in cold climates and highly insulated envelopes. When reviewing these results, it should be noted that though Annual Heating Load (AHL) for the warm climate increased by 30%, these impacts were considered negligible, as the AHL was only 5% of the Annual Cooling Load (ACL). For the same reason, the impacts on ACL in cold climates were also considered negligible, as ACL accounted for only 2-6% of the AHL.

**Table 6.** Difference between steady-state and dynamic analysis for thermal bridges.

Climate	Insulation level	Method	Δ heating load (%)^42^	Δ cooling load (%)^42^	Thermal bridge effect^42^
Warm (Phoenix)	Low	EWM	3 (underestimate)	13 (underestimate)	+30% AHL+20% ACL
		EUVM	8 (under)	17 (under)	
Cold (Quebec)	Low	EWM	9 (under)	4.1 (under)	+18% AHL -24% ACL
		EUVM	13 (under)	26.3 (under)	
	High	EWM	4 (under)	10.4 (under)	+20% AHL -12%ACL
		EUVM	8 (under)	2.7 (under)	

This discussion does not fully convey the added complexity of incorporating energy analysis, whether steady-state or dynamic, into the design process. Energy analysis requires first intimately deducing the envelope’s thermal performance, then analyzing the geometry to understand how airflow moves through the building. Once this is known, the ventilation and temperature control systems can be designed/chosen, which directly impact energy demand based on their sizing and efficiency. The thermal energy performance (i.e., energy demand resulting from the thermal control systems – not total energy demand, as that includes other mechanical systems, lighting, etc.) of the building is dependent on the combination of these factors, along with the building’s usage. Some buildings have time-dependent environmental controls because they do not anticipate occupancy during certain times, thereby reducing heating/cooling loads. These factors combine to add multiple layers of complexity to the already complex, iterative process. However, since codes and standards impose limitations on energy demand and intensity, some energy analysis is required to ensure conformity.

To achieve more accurate, dynamic thermal and energy analysis, the analysis must be conducted. This has a few drawbacks, including the need for extremely large environmental datasets and the high computational demand. On top of complex computations covering many aspects (e.g., thermal energy flow, fluid dynamics) and the large amount of data being processed, energy analysis simulations should be quite detailed to ensure accurate results, further increasing this demand. This provides a potential future research direction: determining whether a connection between steady-state and dynamic energy analysis can be established, normalized by a climatic factor, so practitioners can easily scale their steady-state results to account for dynamic thermal effects. Such work could substantially improve the speed and practicality with which professionals estimate dynamic thermal effects in design.

### Condensation, moisture, and comfort

Since thermal bridges locally increase heat flow, interior surface temperatures at these locations fall below those of surrounding areas during the heating season. If the surface temperature drops below the indoor air dew point, condensation occurs, leading to damp patches, staining, and the potential for mould growth.^13,14^ Moisture accumulation can degrade finishes, reduce insulation effectiveness, and in some cases affect structural components, compromising durability and indoor air quality.^11^ Many design guidelines, therefore, specify minimum interior surface temperatures at critical locations to prevent mould. Junctions with high Ψ require careful insulation and air sealing to maintain surface temperatures above the dew point. Figures 6 and 7 (Section 4.5) illustrate how adding insulation around corners raises interior surface temperatures and eliminates condensation risk.

It has been noted that thermal bridges are more critical in cold climates, since the magnitude of heat flow is proportional to the temperature difference, which is greater there. However, this only considers consequences on energy demand. In warm, humid climates, such as those in the Mediterranean, thermal bridging can be a major issue even with small temperature differences. The contrast between the warm, humid environment and the climate-controlled environment heavily promotes condensation. This observation underscores that thermal bridging is not just an energy-efficiency issue. Additionally, as the temperature rises, the dew point tends to rise, further increasing the risk of condensation.

Thermal bridges also influence thermal comfort. Cold interior surfaces increase radiant heat loss from occupants, who may feel discomfort even when the air temperature is set to the setpoint. Mitigating major bridges helps maintain more uniform surface temperatures, reducing discomfort effects and improving perceived comfort, while reducing local draughts near cold walls or floors.

### Economic impacts and payback

Evola et al.^44^ investigated energy and economic impacts of thermal bridge mitigation in terraced and semi-detached houses in a Mediterranean climate (Tables 7 and 8). Case B corresponds to the structure with corrected thermal bridges and low levels of insulation, while Case C corresponds to the structure with corrected thermal bridges and high insulation levels. Correcting thermal bridges by insulating corner pillars and slab edges reduced heating loads by roughly 17–25% and cooling loads by about 3–4%, translating to annual energy savings of approximately 8–10% and similar reductions in CO_2_ emissions. However, because the climate is relatively mild, the absolute energy savings were modest, and payback periods for retrofit measures were long: around 18–20 years in some terraced house scenarios and not reached within 25 years for certain semi-detached cases. In colder climates, where heating demands and energy costs are higher, the same measures would be expected to yield larger absolute savings and shorter payback times. These findings underscore that the economic attractiveness of thermal bridge mitigation depends on climate, energy costs, and building typology. However, they also show that, even in moderate climates, there is a non-negligible energy and emissions benefit.

**Table 7.** Energy impact of thermal bridge mitigation in a Mediterranean climate.

House type	Case	Heating load decrease^44^	Cooling load decrease^44^	Annual energy savings^44^	CO2 emission decrease^44^
Terraced	Case B	25.2%	3.0%	8.3%	8.0%
	Case C	24.6%	2.7%	7.9%	7.6%
Semi-detached	Case B	18.4%	4.2%	9.7%	9.5%
	Case C	16.9%	3.7%	8.9%	8.7%

**Table 8.** Economic analysis of thermal bridge mitigation.

House type	Case	Annual energy bill savings %^44^	Construction cost increase %^44^	Retrofit cost increase %^44^	Payback period^44^
Terraced	Case B	7.6	6.3	30.0	18 years
	Case C	7.2	6.3	64.1	20 years
Semi-detached	Case B	9.0	14.5	29.5	------
	Case C	8.3	16.0	68.7	------

## Mitigation of thermal bridges

Mitigation strategies aim to either interrupt conductive heat paths or enhance insulation at critical locations and should be applied in a coordinated manner across the building envelope. This section provides an overview of the mitigation strategies for different locations and causes of thermal bridging.

### Openings

**Figure 8.**
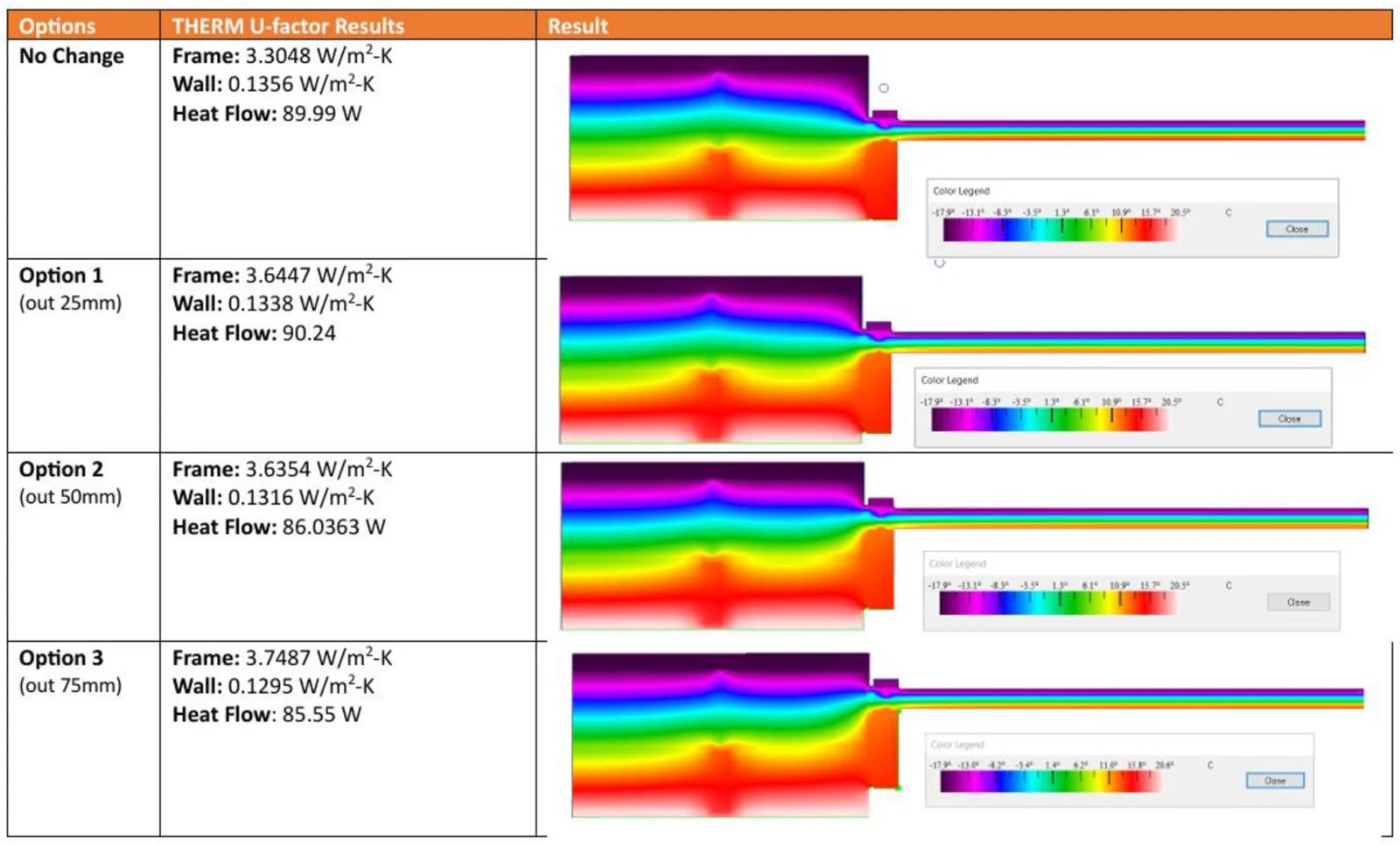
Window placement options relative to the insulation plane (Option 1 – fully inset, Option 3 – aligned with exterior insulation. Option 3 had the lowest heat flow).

Mitigating thermal bridges around windows and doors relies on the combination of high-performance components and proper integration with the surrounding wall. Due to the nature of the opening, increasing insulation levels in/around the opening or using materials with better thermal properties are among the only strategies to reduce the impact of thermal bridging. Heating systems in or around openings seldom reduce energy demand, as they require energy to operate. High-performance glazing (double- or triple-pane with low-emissivity coatings and inert gas fills) reduces glass U-values, vastly reducing heat flow and thermal bridging through the frame. In contrast, frames made from wood, uPVC, fibreglass, or aluminum with effective thermal breaks reduce conduction through the frame.^18,19^ Insulated frames and spacers, such as foam-filled chambers or insulated PVC profiles, further improve frame performance, as demonstrated by Smusz et al.^20^ Continuity of insulation at the perimeter is crucial: extending or wrapping insulation around jambs, sills, and heads links the window or door assembly thermally to the wall insulation and can significantly reduce perimeter Ψ-values.^11^ Optimal window placement, with frames aligned in the insulation plane (Figure 8), minimizes the length of conductive paths and results in lower heat flow than fully inset configurations. Robust air and moisture sealing using appropriate foams, membranes, and sealants is required to avoid convective bypass and moisture ingress.^17^ When these measures are combined, thermal bridge effects at openings can be reduced to the point where interior surface temperatures remain close to those of the adjacent wall, even in cold climates. As stated in Section 3, quality assurance can play a significant role in mitigating window thermal bridges.^17,20^ It is crucial that no gaps in insulation or misalignment of elements arise during installation, or else the efforts put into designing a thermally efficient envelope will be in vain. Any installation errors exacerbate thermal bridging.

### Exposed elements

**Figure 9.**
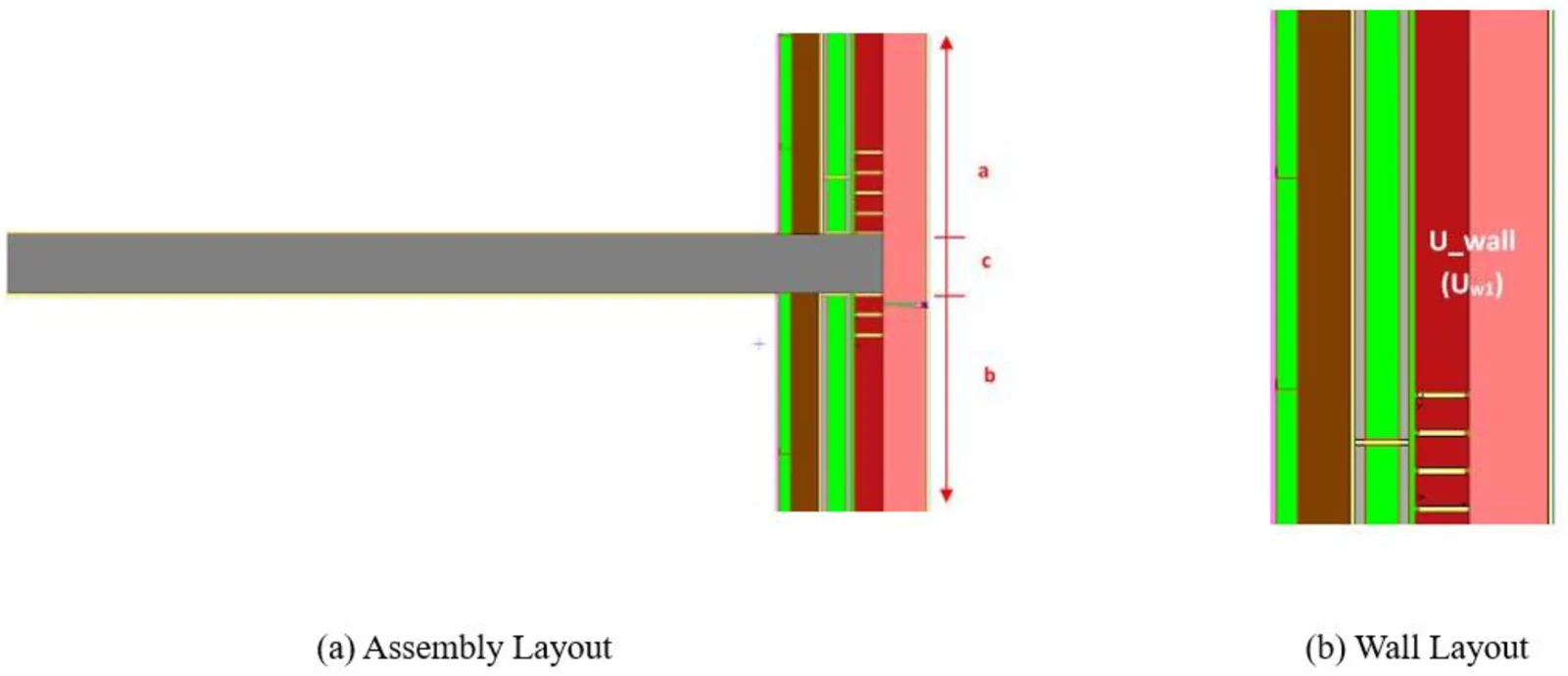
Example detail illustrating slab-edge thermal bridge mitigation with added insulation.

For sunshades, slab edges, columns, and parapets, mitigation generally involves adding thermal breaks at connections, wrapping exposed concrete or steel with insulation, and/or selecting smart materials. Sunshades can be attached using low-conductivity brackets, stainless steel bolts, or purpose-designed thermal-break pads or supported by separate structures that do not penetrate the main insulation layer.^11^ Slab edges can be addressed by recessing slabs to allow continuous exterior insulation or by applying insulation over the exposed edges and soffits (Figure 9). The clear wall R-Value, considering the added insulation, is about R-48.5. The presence of the slab edge reduces this R-value to approximately R-35.5. However, without the added insulation, the slab edge would reduce the R-value to R-24.5. This example demonstrates the impact of slab-edge thermal bridges and shows that even thin layers of added insulation can significantly reduce Ψ.^23^ Exposed columns may be enclosed in insulated cladding or jackets; Evola and Gagliano^22^ showed that adding 6–14 cm of EPS around corner columns increased interior surface temperatures by about 2°C, reducing condensation risk. However, the relative share of heat loss through the column rose as the surrounding walls were insulated. Parapets and roof edges should be detailed so that roof insulation continues up and over the parapet or so that parapets are constructed as separate, fully insulated assemblies, avoiding thermal “short circuits” at the roof–wall junction.

### Walls

Mitigating thermal bridges in walls involves both selecting a framing system and using continuous insulation. Where possible, wood framing is preferred over steel because of its lower conductivity. Where steel is required, thermal break strips between studs and sheathing, staggered stud layouts, or engineered studs with perforations can reduce conductive cross-sections. Continuous insulation layers on the exterior or interior of framed walls thermally isolate studs and ties and are a core strategy in high-performance envelopes.^11^ Connectors for cladding, such as brick ties, shelf angles, and clips, should use lower-conductivity materials (e.g., stainless steel, fibreglass, or plastic) and thermally broken support systems.^13^ In insulated sandwich panels, using low-conductivity ties, minimizing steel connectors, and avoiding uninsulated solid regions around openings can significantly improve effective R-values.^24^ In retrofits, adding exterior insulation or insulated interior linings can substantially reduce thermal bridging through walls, even in existing masonry or concrete buildings.

### Wall-to-wall or roof connections

At corners and junctions, the primary objective is to maintain continuous insulation across the connection. External insulation systems must be detailed so that boards or panels wrap around corners without gaps or misalignments. Wall and roof insulation should meet or overlap at eaves and parapets; uninsulated concrete upstands or gaps at the roof–wall interface should be avoided. Where external insulation is not feasible, thin internal insulation angles or strips applied at wall–wall and wall–ceiling junctions, as described by Lohmann,^45^ can raise interior surface temperatures and reduce bridging. Only in exceptional cases where insulation cannot be added and condensation is severe, have low-power heating elements been used along junctions to maintain temperatures above the dew point; such measures are energy-intensive and generally considered a last resort.

### Balconies

Balconies pose one of the most challenging thermal bridge problems due to structural demands and occupant expectations. Eliminating or reducing balcony projections, for example, by using Juliet balconies or separate free-standing balcony structures, is the most direct way to avoid penetration of the insulation layer. Where balconies are required, discrete supports such as steel brackets or posts, especially when combined with thermal break plates at connection points, can transform a continuous linear bridge into a series of less severe point bridges. Häberli and Collins^26^ showed that such discrete connection systems can substantially reduce Ψ and increase effective R-values (Table 3). STB modules installed between interior floor slabs and exterior balcony slabs are now widely used; these devices combine low-conductivity (occasionally high-strength) insulation with stainless steel or FRP reinforcement and can reduce Ψ by 50–90%, significantly warming interior slab edges.^26,32^ However, high product costs and low industry awareness are active barriers to the widespread adoption of STBs. Wrapping existing balconies with insulation on the underside and exposed edges is a practical retrofit measure that, while less effective than structural breaks, nonetheless reduces heat loss and improves surface temperatures. In all cases, detailing around balcony doors, thresholds, waterproofing, and drainage must be coordinated with thermal measures to avoid moisture problems and ensure structural performance.

### Discussion

Though each thermal bridging location has its own nuances, many commonalities can be seen between mitigation strategies. At a basic level, mitigation comes down to either increasing the thermal properties of the bridging element (through material selection, or strategies like insulation wrapping that artificially increase thermal properties) or ensuring the insulation continuity of the building envelope is retained (such as by designing elements to minimize penetration or using STBs in penetrative zones). Often, the best strategies combine these strategies by designing systems that minimize the penetration of the building envelope and replace penetrative materials with those with better thermal properties, such as GFRP cladding attachment systems, or by combining discretely connected balconies through STB systems. Also, at multiple bridging locations, it has been shown that installing an energy-powered heating system does not improve the structure’s overall energy performance. The only case in which such a system should be used is when moisture-related impacts are of concern, and specific areas require staying warm and dry, with no better alternative. However, the decision-making behind thermal bridging mitigation and the design of structures seldom comes down to the best performance.

As discussed in Section 5, designing a structure and mitigating thermal bridging is an iterative process that requires many simulations and consideration of many alternative solutions. When deciding between strategies, the overall benefits in terms of thermal and energy performance are weighed against factors such as cost, material availability, and industry knowledge. Which materials are available and which strategies/techniques workers are comfortable or experienced with will depend on the location where the structure is being designed/built. Cost is one of the most impactful metrics when making these decisions. The strategies employed will heavily affect costs, both through material costs and through increased project schedules and labour costs.

Additionally, someone must pay designers for the lengthy, iterative design process mentioned throughout the review. The more detailed the design process, the longer it will take. Mitigating thermal bridging in different areas will incur different costs. The more common the element is in the structure, the more total pieces that need to be changed. Some areas are just more expensive due to specific strategies that create detriments, such as mitigating balcony thermal bridges. Using a continuous balcony with an STB simplifies construction. However, it incurs far higher product costs than discretely connected balconies, which require a more complex and expensive installation process, both of which are among the more expensive thermal bridge mitigation solutions.

All of this is to say that thermal bridge evaluation and mitigation are not simple. The evaluation process is long, iterative, and dependent on a massive quantity of factors, making it extremely hard to create a generalized framework. The choice of mitigation strategy is equally complex, since the required elements can be climate-dependent (e.g., sunshades vary with climate), and decisions to mitigate one section affect the performance of all surrounding sections. In practice, fully mitigating every thermal bridge in a structure is rarely economical; therefore, designers must typically prioritize the details with the greatest effect on envelope performance, moisture risk, and constructability. By assessing many thermal bridge mitigation strategies, it can be seen that there is still room for improvement in terms of performance, constructability, and the integration of thermal and structural systems. Such aspects will be expanded upon in the following section.

## Research needs and future directions

Despite substantial advances in understanding and mitigating thermal bridging, several important gaps remain, including the limited breadth of experimental data, the lack of long-term in-situ monitoring, and the need for expanded studies on the application and generalization of analysis methodologies.

Firstly, there is a major need to expand the body of experimental data. Much of the current knowledge is based on numerical simulations, while full-scale laboratory experiments and in-situ measurements on real buildings remain comparatively limited.^43^ Additional guarded hot-box tests and field monitoring of typical junctions—such as wall–slab, wall–column, balcony–wall, and window interfaces—would help validate and refine calculation methods (EWM, EUVM, Ψ/χ catalogues) and improve confidence in design tools.^41,46^ This issue likely stems from the complexity of such experiments; thermal bridging is best measured by concurrently exposing one element to multiple environmental conditions, which can be energy-intensive and expensive. This is especially true for structural components, such as balcony thermal bridge mitigation tools, whose role in load-bearing and structural stability necessitates an additional layer of testing to ensure product safety.

There is also scope for innovative materials and integrated solutions that offer structural capacity with low thermal conductivity. High-strength FRPs, engineered timber systems, aerogel blankets, vacuum-insulated panels, and hybrid components could enable details with minimal thermal bridging while meeting structural, fire, and serviceability requirements. A promising area of future work is investigating other properties of these materials/assemblies to aid in developing multifunctional elements that combine structural, thermal, and other roles (e.g., seismic fuses, fire barriers).

Another goal practitioners should have is understanding long-term in-situ performance. Many thermal break products, high-performance windows, and advanced insulation technologies have only been in widespread use for a few decades, so their performance under prolonged exposure to moisture, freeze–thaw cycles, and mechanical loads is not fully documented. Long-term monitoring of buildings that incorporate these technologies would clarify degradation mechanisms, maintenance needs, and actual performance over time.

The standardization and development of design tools are interesting and important potential research directions. While standards such as ISO 14683^36^ and national guidelines provide some Ψ/χ values and modelling rules, comprehensive, regularly updated catalogues covering broader combinations of materials, constructions, and climates would benefit practitioners—frameworks, such as the one created by B.C. Hydro^33^ have been developed for the holistic evaluation of structures, but this process is lengthy and lacks repeatability across future projects. Studies using such a framework could be useful to practitioners in gaining a better understanding of typical results across many building typologies and climates, if such correlations can be made. Similar studies comparing steady-state and dynamic energy analysis would also be useful for establishing such correlations, easing practitioners’ lives and speeding up the design process. Integrating thermal bridge detection and inspection into BIM environments, where 3D models are automatically scanned for insulation breaks and corrective options are suggested, would help mainstream best practices in everyday design.

Finally, emerging systems and configurations—such as façade-integrated photovoltaics, green walls, modular prefabricated envelopes, and new connection systems—introduce novel thermal bridge issues that merit investigation. Coupling technical studies with economic and policy analyses, including life-cycle cost and carbon assessments across climates and building types, would support the evolution of codes and standards to effectively incorporate thermal bridge control as buildings move toward net-zero and ultra-low energy targets.

## Conclusions

Thermal bridging remains a critical challenge in the design and retrofit of energy-efficient buildings. Even in envelopes with high nominal R-values, localized bridges at openings, balconies, corners, exposed elements, and within wall assemblies can significantly increase heat loss, reduce interior surface temperatures, and create the risk of condensation and mould. The relative importance of these bridges grows as codes and standards drive toward higher insulation levels and lower energy use, making their explicit consideration and mitigation indispensable. If design and construction practices do not evolve and new standards are met by simply increasing insulation thicknesses, the impact of thermal bridges will only become more critical. It is prudent for engineers to understand the phenomena that impact buildings’ thermal and energy efficiency so that they can make effective, sustainable design choices.

This review has synthesized current knowledge on the fundamentals and climate sensitivity of thermal bridges, their mechanisms and typologies across different building elements, methods for quantifying their effects (from EWM and EUVM to 2D/3D numerical analysis), and their impacts on energy performance, moisture risk, and comfort, including insights from dynamic simulations and economic studies. It has also outlined practical mitigation strategies and identified key areas where further research and standardization are needed to support widespread adoption of best practices. The main conclusions can be summarized as follows:

• Thermal bridges can account for a substantial portion of total heat loss in modern, well-insulated envelopes and can lead to localized cold surfaces, condensation, mould growth, and reduced durability. Their relative impact increases as overall insulation levels improve, making them a limiting factor in achieving very low energy targets.
• Simple one-dimensional calculations are insufficient to capture thermal bridge effects. Methods such as the Equivalent U-Value Method, supported by reliable Ψ- and χ-values, provide a practical basis for steady-state analysis. Frameworks exist to integrate all thermal bridge effects into a single envelope performance metric while evaluating individual element contributions to thermal losses. At the same time, 2D and 3D numerical tools are often required to capture complex details and assess local surface temperatures. Dynamic simulations show that steady-state approaches tend to underestimate the influence of thermal bridges on annual loads and peak conditions.
• Effective mitigation is available for all major bridge types. High-performance windows and frames, careful placement and insulation at openings, continuous exterior insulation, thermally broken connectors for balconies, cladding, and sunshades, and coordinated insulation at wall–roof and wall–floor junctions can substantially reduce thermal bridging. In many cases, the combination of continuous insulation and targeted STBs (or similar devices, based on the location of thermal bridge) can raise effective envelope R-values and reduce heating energy demand by 10–15% or more.
• The performance of individual details cannot be considered in isolation. The greater the improvement in clear-wall performance, the more critical it becomes to address residual bridges. A holistic, integrated design approach involving architects, structural engineers, and building scientists from the outset is essential to avoid “weak links,” such as unbroken balconies or uninsulated slab edges, which undermine overall performance.
• When designing thermally efficient structures, the process is iterative and time-consuming because many factors interact and decisions are made along the way. Codes and standards do not dictate thermal properties; they only set energy demand and intensity limits, necessitating complex energy models that account for environmental control systems alongside thermal effects. The interactive effects among materials, form, the environment, and ventilation control systems, among other things, make it difficult to determine the most efficient design. In conjunction with limitations on designers, such as cost and material availability, the procedure is highly project-specific, making it difficult to develop an efficient, generalized framework for evaluating and designing sustainable, energy-efficient structures.
• Further experimental validation, the development of innovative low-conductivity structural solutions, long-term in-situ performance monitoring, and expanded standardized Ψ/χ catalogues are needed to support robust design and code evolution. Integration of thermal bridge checking and optimization into BIM and mainstream design workflows will be a key step toward translating best practices into everyday construction.

In conclusion, minimizing thermal bridging is a prerequisite for achieving high-performance, low-energy, and durable buildings. A holistic approach is required for performing such analysis and design, due to the interactive nature of all factors affecting the thermal and energy efficiency of buildings. Current knowledge, tools, and products already allow the near elimination of major thermal bridges in new construction and significant reductions in existing buildings through well-planned retrofits. Ensuring that thermal bridge analysis and mitigation are integral to the design process, rather than optional refinements, is central to realizing buildings that perform as intended in terms of energy efficiency, comfort, and long-term resilience.
